# Reliability of a new test battery for fitness assessment of the European Astronaut corps

**DOI:** 10.1186/s13728-015-0032-y

**Published:** 2015-08-14

**Authors:** Nora Petersen, Lutz Thieschäfer, Lori Ploutz-Snyder, Volker Damann, Joachim Mester

**Affiliations:** Wyle GmbH, Cologne, Germany; Space Medicine Office (HSO-AM), European Astronaut Centre Department (ESA), Directorate of Human Spaceflight and Operations (D/HSO), European Space Agency, Geb. 12, Linder Höhe, PO Box 906096, 51147 Cologne, Germany; Institute of Physiology and Anatomy, German Sport University Cologne (DSHS), Am Sportpark Muengersdorf 6, 50955 Cologne, Germany; Universities Space Research Association, NASA Johnson Space Center, B261, SK3, Houston, TX 77058 USA; Institute of Training Science and Sport Informatics, German Sport University Cologne (DSHS), Am Sportpark Muengersdorf 6, 50933 Cologne, Germany

**Keywords:** Astronaut fitness assessment, Space flight, Test battery, Reliability, Intraclass correlation coefficients (ICC)

## Abstract

**Background:**

To optimise health for space missions, European astronauts follow specific conditioning programs before, during and after their flights. To evaluate the effectiveness of these programs, the European Space Agency conducts an Astronaut Fitness Assessment (AFA), but the test–retest reliability of elements within it remains unexamined. The reliability study described here presents a scientific basis for implementing the AFA, but also highlights challenges faced by operational teams supporting humans in such unique environments, especially with respect to health and fitness monitoring of crew members travelling not only into space, but also across the world. The AFA tests assessed parameters known to be affected by prolonged exposure to microgravity: aerobic capacity (*V*O_2max_), muscular strength (one repetition max, 1 RM) and power (vertical jumps), core stability, flexibility and balance. Intraclass correlation coefficients (ICC_3.1_), standard error of measurement and coefficient of variation were used to assess relative and absolute test–retest reliability.

**Results:**

Squat and bench 1 RM (ICC_3.1_ = 0.94–0.99), hip flexion (ICC_3.1_ = 0.99) and left and right handgrip strength (ICC_3.1_ = 0.95 and 0.97), showed the highest test–retest reliability, followed by *V*O_2max_ (ICC_3.1_ = 0.91), core strength (ICC_3.1_ = 0.78–0.89), hip extension (ICC_3.1_ = 0.63), the countermeasure (ICC_3.1_ = 0.76) and squat (ICC_3.1_ = 0.63) jumps, and single right- and left-leg jump height (ICC_3.1_ = 0.51 and 0.14). For balance, relative reliability ranged from ICC_3.1_ = 0.78 for path length (two legs, head tilted back, eyes open) to ICC_3.1_ = 0.04 for average rotation velocity (one leg, eyes closed).

**Conclusions:**

In a small sample (*n* = 8) of young, healthy individuals, the AFA battery of tests demonstrated acceptable test–retest reliability for most parameters except some balance and single-leg jump tasks. These findings suggest that, for the application with astronauts, most AFA tests appear appropriate to be maintained in the test battery, but that some elements may be unreliable, and require either modification (duration, selection of task) or removal (single-leg jump, balance test on sphere) from the battery. The test battery is mobile and universally applicable for occupational and general fitness assessment by its comprehensive composition of tests covering many systems involved in whole body movement.

## Background

Assessments of physical fitness are frequently used in occupational health care settings to determine an individual’s suitability to operate in a specific environment and their capacity to perform required occupational activities without risk to their health and safety, or that of their colleagues [1, 2].

When performed periodically and systematically, these assessments may help identify small changes in an individual’s physical condition that could compromise their performance and/or ability to work safely, which can then be addressed with remedial action. Physical fitness assessments with European Space Agency (ESA) astronauts are performed to objectively quantify physical performance changes after return from space flight. To increase the quality of the data produced and support both operational and research needs, the former simple, gym-based testing protocol was replaced by the ESA’s European Astronaut Centre (EAC) Astronaut Fitness Assessment (AFA), a broader, instrumented test battery. An additional consideration is that the AFA setup must be mobile, as ESA astronauts returning from the International Space Station (ISS) may need to be tested away from ESA facilities, both in the United States and Russia, where post-flight rehabilitation is sometimes implemented. As such, not only must the test elements assess systems affected by space flight and comply with sport scientific evaluation standards, but the test equipment must also be portable and the assessment procedures implementable in various gym environments. This requires a simple test setup, but one that is still capable of producing meaningful data under “field testing” conditions, rather than the standard laboratory conditions available at EAC.

Ten individual tests are included in the AFA. These consider astronauts’ unique occupational performance profile, which is characterised by specific tasks and environmental factors, such as launch and landing, extra-vehicular activities (space ‘walks’) and ISS-specific operations whilst being exposed to microgravity (µG), and ultimately the return into the Earth’s gravity. Microgravity exposure for up to 6 months is known to induce decreases in muscle strength [3, 4], bone mineral density [5, 6], cardiovascular endurance [7, 8] and postural control [9–12], and the AFA tests are included based on this current knowledge: anthropometry (height, body mass, and body composition), hip flexibility, handgrip strength, balance, posture and gait characteristics, core, lower and upper body muscle strength, vertical jump (muscular power) and cardiovascular capacity.

A further difficulty in the development of the AFA test battery is the lack of a precise definition of the physical occupational demands of spaceflight. However, although test validity in relation to space flight occupational performance cannot be assessed at this time, the reliability of the new test battery can and should be assessed. This has not been performed previously, because EAC’s remit is to provide operational support to ESA astronauts and, historically, it has not had the resources to perform research activities. In addition, the test battery was established for organizational reasons (i.e. an increasing number of ESA long-duration space missions and increased independence of ESA from the other ISS Partners) and the battery was developed and used in parallel to operational implementation, and has undergone numerous changes in the course of development.

Towards this end, the purpose of this investigation was to report the reliability (retest correlation, systematic bias and random error) of each test element, to support the decision to keep, modify or remove them from the AFA.

## Methods

### Participants

Ten male subjects were recruited to participate in the study. The inclusion criteria were based on anthropometric selection standards for ESA Astronauts: healthy and matching the astronaut population in terms of body height (between 149.5 and 190.5 cm) and body mass (≤95.0 kg) [13]. The study was approved by the ethical board of the German Sport University in Cologne and all subjects provided informed written consent before participation.

### Study design

The study used a test–retest design in a controlled laboratory environment, with participants making three visits to the German Sport University, with each visit separated by 7 days. Prior to the first experimental visit, participants performed a familiarisation session of the entire test battery. For experimental visits, participants arrived at approximately the same time of day, wearing the same clothes and shoes for each visit. They were instructed to not deviate from their usual training and eating habits during the testing period. To minimise measurement errors, subject position, movement speed, observer instructions, measuring instrument, location and test conditions were standardised between sessions.

The test elements were always implemented in the same order, with the aim of minimising fatigue effects (e.g. elements with a low physical demand were scheduled at the beginning of the battery prior to implementation and those requiring significant/maximal physical effort at the end) with 1–3-min rest breaks between measurements and, as with the AFA performed with ESA astronauts, all elements were completed in a 2-h time period. Consistent with normal AFA procedures, subjects ran on a treadmill for 10 min at 10 km h^−1^ to warm up and no other specific warm-up exercises were completed. To avoid observer bias, all experimental staff were familiarised with the tests to which they were assigned and they conducted these tests for the entire study.

#### Anthropometry

Height was measured using a stadiometer (SECA GmbH, Hamburg, Germany). Body mass was measured and percentage body fat estimated using a combined weighing scale and bio-electrical impedance device (BC-418 MA, Tanita, Tokyo, Japan).

#### Flexibility

Hip flexion was measured with a Sit-and-Reach box (Sport Time, USA). Participants were instructed to reach forward as far as possible in a slow and controlled movement and hold the final position for 2 s. The distance (in cm) achieved was measured and three trials were performed, with the single best effort used for analysis.

Hip extension was measured using a modified Thomas Test [14]. Participants adopted a supine position on a bench with both legs bent over the edge. Allowing the measured leg to hang freely, participants were instructed to pull the other knee to their chest ensuring continuous firm contact of the lumbar spine with the bench surface. Hip angle (°) in relation to the bench surface was measured using an inclinometer (ACU 360, Lafayette Instrument Company, Lafayette, USA) at the mid-thigh, capturing six consecutive values in the same position. The average of those six values was used for analysis. An identical measurement was then made with the other leg.

#### Handgrip strength

Maximal, one-handed handgrip strength was measured for both hands using a mechanical handgrip dynamometer (Takei Scientific Instruments Co. Ltd., Niigata City, Japan). From a standing position, with their arm down by their side, participants were instructed to apply maximal force for 2 s. Participants made three attempts per hand, alternating hands each time, separated by at least 60 s rest, with the single best effort used for analysis.

#### Core strength

The ability to maintain a standardised position and movement was measured in three different (ventral, lateral and dorsal—in that order) positions as described in the Swiss Olympic manual of core strength assessment [15]. In each position, participants were requested to maintain both position and speed of movement (1 Hz) in synchronisation with a metronome (Ma-30, KORG metronome, Tokyo, Japan). The test was terminated when the subject was unable to maintain the required position or movement [15] after either a maximum of two warnings by the test observer or until volitional fatigue. The time (in s) to test termination was recorded in all three positions.

#### Muscle strength

Muscle strength was assessed by estimating the one repetition maximum (1 RM) using the Brzycki Formula [16, 17]. Bench press and squat manoeuvres were conducted in a standardised body position (feet, hands, and bench) and range of motion in relation to the rack (Smith machine, gym80, International GmbH, Gelsenkirchen, Germany). Participants were instructed to perform as many repetitions as possible at a pre-selected load with the aim of achieving volitional fatigue in less than 10 repetitions.

#### Balance

**Fig. 1:**
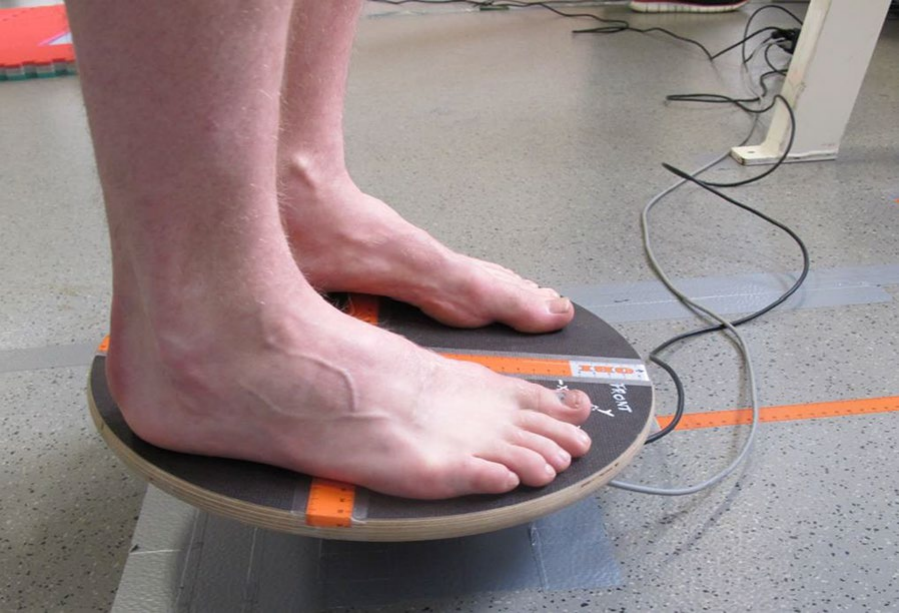
Instrumented balance board used for balance tasks (L7–10).

Ten tests of balance were performed using two different instruments (Table 1). To assess postural sway area of the body’s centre of pressure (COP) and COP displacement path length, six tests (Levels 1–6), each with an increasing level of difficulty, were performed on a pressure distribution platform (FDM-S Pressure Plate, Zebris Medical GmbH, Isny, Germany). Data were processed at 100 Hz using Zebris software, with COP area taken as the area (in mm) within the 95 % confidence interval. The last four tests (Levels 7–10) were performed on a balance board (Fig. 1) with a metal spherical base (Sport Thieme GmbH, Grasleben, Germany) instrumented with an inclinometer (BalensoSenso, Fa. Reinert, Pforzheim, Germany) inserted into the sphere underneath the board to measure angular velocity.

**Table 1:** Balance test Levels 1–10, implemented on pressure plate and balance board

Difficulty level	Test conditions
Pressure plate	Tasks
Level 1	Both feet, eyes closed
Level 2	One foot, eyes open
Level 3	One foot, eyes closed
Level 4	Both foot, eyes open, head tilt
Level 5	Both feet, tip toes, eyes open
Level 6	Both feet, tip toes, eyes closed
Balance board	
Level 7	Both feet, eyes open
Level 8	One foot, eyes open
Level 9	Both feet, eyes closed
Level 10	One foot, eyes closed

Test conditions with both devices were made increasingly difficult by closing the eyes, standing on one foot, tilting the head back and standing on tiptoes, which was a modification of existing balance tests in rehabilitation practice [18]. Foot and hand positions were standardised (hands on hips, surface markers for feet) and tests on one foot were always performed with the same leg. All tests lasted a maximum of 15 s. Stepping off the device surface, the hands losing contact with the hips (e.g. to grasp safety handles) and opening the eyes (for tests with eyes closed) were termination criteria for any test. In the case of termination, the maximum time achieved by the subject was recorded. All ten tests were completed in the same order, regardless of the subject’s ability to complete the full 15 s for any test.

#### Muscle power

Lower body muscle power was assessed from a countermovement jump (CMJ), squat jump (SJ), single-leg CMJ jumps [right (SLJ-R) and left (SLJ-L) leg] and a drop jump (DJ) from a 0.28-m platform. In bare feet, participants were instructed to jump as high as possible whilst keeping their hands in contact with their hips at all times. Each jump was attempted three times, with a break of 60 s between jumps and the single best effort for each task was used for analysis. Performance was measured by calculating jump height (m) based on measurement of GRF (N), contact time (s) and rate of force development (RFD) (N/s) were and using a force platform (5691 A, Kistler, Winterthur, Switzerland) and analysis software (TEMPLO© by Contemplas GmbH, Kempten, Germany) with a sampling rate of 300 Hz [19]. Reactive strength index (RSI) was also calculated for the drop jump as a measure of stretch–shortening cycle function.

#### Aerobic capacity

**Fig. 2:**
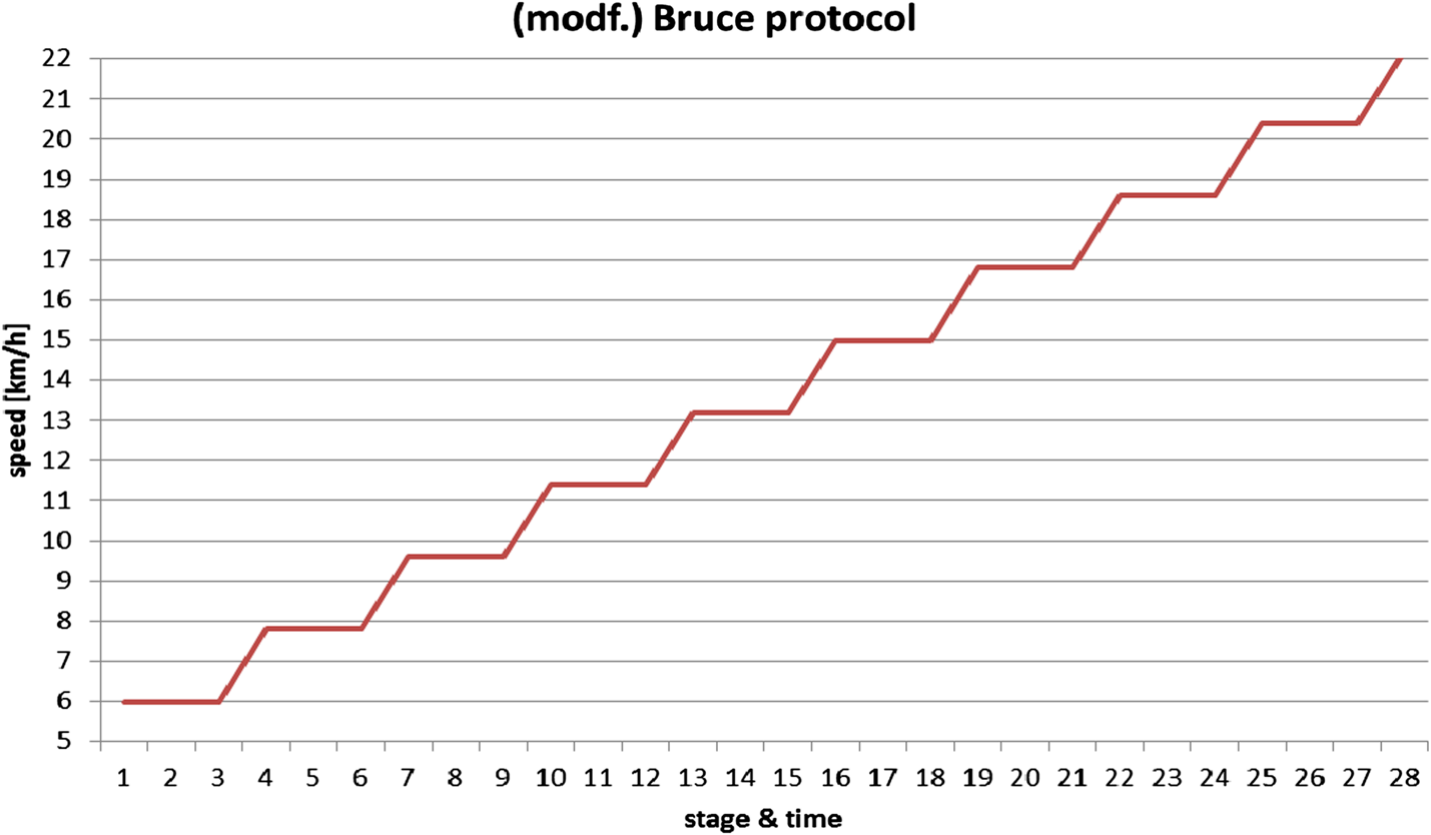
Treadmill protocol (modified Bruce protocol) used for the assessment of aerobic capacity [speed (km h^−1^); stage time (min)].

Aerobic capacity (*V*O_2max_) was measured on a treadmill (PPS 55med-I, WOODWAY GmbH., Weil am Rhein, Germany) using a modified Bruce protocol [20] (Fig. 2). Belt speed was increased by 1.8 km h^−1^ every 3 min (starting at 6 km·h^−1^) at a constant 1 % incline until volitional fatigue, with 30-s breaks between intervals for lactate sampling (“Lactate scout”, EKF-diagnostic GmbH, Magdeburg, Germany).

Oxygen uptake was measured continuously using a spirometry system (Zan600, ZAN Austria e.U., Steyr-Dietach, Austria) and *V*O_2max_ calculated from a sliding mean over the last 30 s before fatigue. Heart rate was recorded using a chest strap and watch (RS800, POLAR, Kempele, Finland). Earlobe lactate samples were taken 1 and 5 min after the point of fatigue, and all lactate values inserted into the ERGONIZER^®^ software (ERGONIZER^®^ version 4.1.10, Kai Röcker, Freiburg, Germany) to provide a secondary estimation of *V*O_2max_. In the AFA performed with astronauts, this estimation technique is used when the spirometry equipment is not available to make a direct measurement.

### Statistical analysis

Data are reported as mean ± 1 SD unless otherwise stated. The main objectives of the assessment were to evaluate relative (Intraclass correlation coefficients, ICC_3.1_) with fixed raters, and absolute (standard error of measurement, SEM, and coefficient of variation, CV) reliability of each element. The rationale for the fixed raters was that, in the operational implementation of the AFA, an individual astronaut is always tested by the same person for consecutive AFAs, and thus, in this study, the same operators always conducted specific test elements and inter-rater correlations were not assessed.

Data from the three experimental visits were analysed using a repeated measures analysis of variance (ANOVA) (*k* = 3; *α*-level = 0.05) to calculate SEM, ICC_3.1_, and the *F*-ratio, to identify systematic bias (critical *F* value >3.74) potentially caused by implementation and analysis procedures, learning and/or fatigue effects [21–23]. Prior to analysis, data were tested for normal distribution and homoscedasticity, and, where not evident, a transformation was applied. Thus, a log100 transformation was applied to the following data: *balance: COP* sway area (Level 1, 2, 3, 5 and 6), path length (Level 4 and 5), and average rotation velocity (Level 7 and 8); *jump*: CMJ (height and RFD), SJ (height and RFD), SLJ (RFD for both legs jump height for SLJ-L) and DJ (RFD); *VO*_*2max*_: estimation by ERGONIZER^®^; *core strength*: dorsal position. The measures of error (SEM, CV) are reported in absolute form (‘+/−’), or in ratio form (‘×/÷’) for log100 transformed data. Normal distribution or homogeneity, although statistically tested here, may still differ for a larger sample and, therefore, both SEM and CV are always provided. Statistical analysis was performed with commercially available software (PASW Statistics 18, IBM Corporation, Armonk, USA) and “Microsoft Excel 2013” (Microsoft, Redmond, USA).

## Results

Of the ten participants who were recruited into the study, only eight [(mean ± 1 SD) age 25 ± 2 years; height 1.78 ± 0.05 m; body mass 76.6 ± 8.6 kg] completed all the required procedures and were thus included in the statistical analysis.

### Anthropometry

Relative reliability for anthropometric parameters were: body mass: ICC_3.1_ = 0.99; SEM = 0.73 kg; height: ICC_3.1_ = 0.99; SEM = 0.23 cm and % body fat ICC_3.1_ = 0.89; SEM = 1.80 % (Table 2). No systematic error was detected with the *F* test.

**Table 2:** Relative and absolute reliability of anthropometry and flexibility measures

Parameter	Pooled mean ± SD	Relative reliability	Absolute reliability	
		ICC_3.1_	SEM	CV
Body mass (kg)	76.8 ± 9.7	0.99	±0.73	±0.95
Height (cm)	180.0 ± 5.2	0.99	±0.23	±0.13
Body fat (%)	11.4 ± 5.3	0.89	±1.80	±15.81
Sit-and-Reach (cm)	27.3 ± 9.8	0.99	±1.20	±4.41
Thomas Test right (°)	23.1 ± 7.0	0.85	±2.69	±11.63
Thomas Test left (°)	24.8 ± 6.7	0.63	±4.12	±16.63

*SD* standard deviation, *ICC* intraclass correlation coefficient, *SEM* standard error of measurement, *CV* coefficient of variation.

### Flexibility

Hip flexion (Sit-and-Reach test) showed a correlation of ICC_3.1_ = 0.99; SEM = 1.20 cm, with hip extension (Thomas Test) showing a correlation of ICC_3.1_ = 0.85; SEM = 2.69° (right leg) and ICC_3.1_ = 0.63; SEM = 4.12° (left leg) (Table 2).

### Muscle strength

Handgrip strength (right and left hand) showed correlations of ICC_3.1_ = 0.97, SEM = 1.96 kg and ICC_3.1_ = 0.95; SEM = 2.50 kg, but also revealed a systematic error in the *F* test (*F* = 7.10, *P* = 0.01) for the right hand only across all trials.

**Fig. 3:**
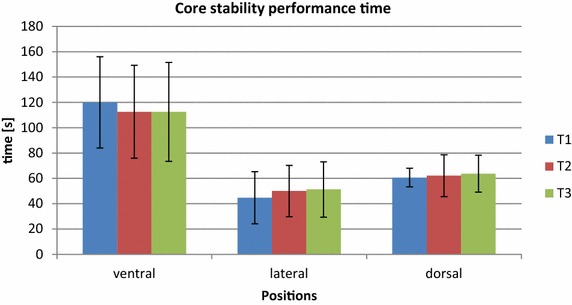
Mean (+SD) core strength test performance time (s) for the ventral, lateral and dorsal position on the three test days.

Time to termination in the core strength tests showed correlations of ICC_3.1_ = 0.89; SEM = 12.66 s and ICC_3.1_ = 0.86; SEM = 7.90 s for the ventral and lateral positions. Data from dorsal position demonstrated a lower correlation of ICC_3.1_ = 0.78; SEM = 1.11 s (Table 3; Fig. 3).

**Table 3:** Relative and absolute reliability of handgrip, core, and squat and bench press strength

Parameter	Pooled mean ± SD	Relative reliability	Absolute reliability	
		ICC_3.1_	SEM	CV
Ventral (s)	115.0 ± 37.4	0.89	±12.66	±11.00
Lateral (s)	48.6 ± 21.0	0.86	±7.90	±16.24
Dorsal (s)	62.2 ± 13.5	0.78	×/÷1.11	×/÷10.71
Handgrip strength right (kg)	50.8 ± 10.6	0.97	±1.96	±3.87
Handgrip strength left (kg)	48.0 ± 10.7	0.95	±2.50	±5.22
1 RM bench press (kg)	87.3 ± 24.0	0.99	±2.48	±2.84
1 RM squat (kg)	135.5 ± 35.9	0.94	±8.7	±6.4

*1 RM* one repetition max, *SD* standard deviation, *ICC* intraclass correlation coefficient, *SEM* standard error of measurement, *CV* coefficient of variation, “±” error in absolute form, “×/÷” error as ratio, based on log transformed data.

The 1 RM strength estimate tests showed correlations of ICC_3.1_ = 0.99; SEM = 2.48 kg for bench press and ICC_3.1_ = 0.94; SEM = 8.67 kg for squat (Table 3). A systematic error was identified with the *F* test [6.18 (*P* = 0.01)] for squat.

### Balance

Relative reliability ranged from ICC_3.1_ = 0.80 for path length on Level 4 on the pressure plate (both legs, head tilted back, eyes open) to ICC_3.1_ = 0.04 for average rotation velocity in Level 9 on the balance board (Table 4). Measurement precision indicated error ranges between 18.2 % (CV of COP path length Level 2) and 89.7 % (CV of COP sway area Level 6).

**Table 4:** Relative and absolute reliability of balance parameters

Parameter	Pooled mean ± SD	Relative reliability	Absolute reliability	
		ICC_3.1_	SEM	CV
Sway area L1 (mm^2^)	22.5 ± 12.6	0.07	±12.20	±54.33
Sway area L2 (mm^2^)	114.8 ± 58.2	0.57	×/÷1.37	×/÷36.75
Sway area L3 (mm^2^)	894.2 ± 882.0	0.58	×/÷1.66	×/÷66.15
Sway area L4 (mm^2^)	45.4 ± 38.3	0.60	±24.24	±53.35
Sway area L5 (mm^2^)	161.3 ± 188.6	0.59	×/÷1.71	×/÷70.75
Sway area L6 (mm^2^)	1,124.0 ± 1,831.1	0.39	×/÷1.90	×/÷89.71
COP path length L1 (mm)	84.8 ± 27.5	0.21	±24.53	±28.94
COP path length L2 (mm)	346.7 ± 98.8	0.59	±63.23	±18.24
COP path length L3 (mm)	1,036.5 ± 433.6	0.63	±263.47	±25.42
COP path length L4 (mm)	92.9 ± 54.2	0.80	×/÷1.25	×/÷24.51
COP path length L5 (mm)	308.7 ± 141.5	0.61	×/÷1.26	×/÷26.37
COP path length L6 (mm)	941.7 ± 327.6	0.62	±202.95	±21.55
Average rotation velocity L7 (°/s)	2.4 ± 1.3	0.54	×/÷1.43	×/÷42.69
Average rotation velocity L8 (°/s)	3.1 ± 1.4	0.55	×/÷1.32	×/÷32.38
Average rotation velocity L9 (°/s)	3.3 ± 1.4	0.04	±1.38	±42.14
Average rotation velocity L10 (°/s)	3.0 ± 1.1	0.35	±0.85	±28.21

*SD* standard deviation, *ICC* intraclass correlation coefficient, *SEM* standard error of measurement, *CV* coefficient of variation, *L* Level, *COP* centre of pressure, “±” error in absolute form, “×/÷” error as ratio, based on log transformed data.

### Muscle power

Correlations ranged from ICC_3.1_ = 0.85; CV = 21.2 % for CMJ RFD, to ICC_3.1_ = 0.14; CV = 14.8 % for SLJ-L height. A systematic error (*F* = 5.09, *P* = 0.02) was only identified for SLJ-R RFD (Table 5). The correlation for drop jump RSI was ICC_3.1_ = 0.73; SEM = 0.15.

**Table 5:** Relative and absolute reliability of jump parameters

Parameter	Pooled mean ± SD	Relative reliability	Absolute reliability	
		ICC_3.1_	SEM	CV
Max flight height CMJ (m)	0.35 ± 0.09	0.63	×/÷1.14	×/÷14.44
Max flight height SJ (m)	0.32 ± 0.06	0.76	×/÷1.10	×/÷9.76
Max flight height SLJ-R (m)	0.15 ± 0.03	0.51	±0.02	±13.09
Max flight height SLJ-L (m)	0.15 ± 0.02	0.14	×/÷1.15	×/÷14.76
Max flight height DJ (m)	0.24 ± 0.06	0.79	±0.03	±11.67
RFD_max_ CMJ (N s^−1^)	11,553 ± 7,275	0.85	×/÷1.21	×/÷21.18
RFD_max_ SJ (N s^−1^)	11,018 ± 5,192	0.74	×/÷1.24	×/÷24.25
RFD_max_ SLJ-R (N s^−1^)	8,634 ± 4,478	0.85	×/÷1.20	×/÷20.10
RFD_max_ SLJ-L (N s^−1^)	8,135 ± 4,324	0.69	×/÷1.32	×/÷31.98
RFD_max_ DJ (N s^−1^)	394,012 ± 240,961	0.65	×/÷1.44	×/÷43.76
RSI (m/s)	1.15 ± 0.29	0.73	±0.15	±13.03

*CMJ* countermovement jump, *SJ* squat jump, *SLJ-R/L* single-leg jump right/left leg, *DJ* drop jump, *RFD* rate of force development, *RSI* reactive strength index, *SD* standard deviation, *ICC* intraclass correlation coefficient, *SEM* standard error of measurement, *CV* coefficient of variation, “±” error in absolute form, “×/÷” error as ratio, based on log transformed data.

### Aerobic capacity

**Fig. 4:**
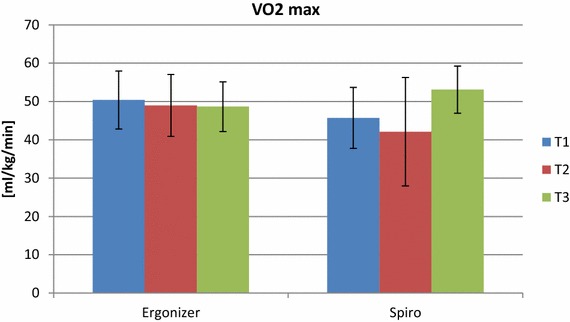
Mean (+SD) measured (via spirometry) and estimated (from blood lactate samples and using the ERGONIZER^®^ software) aerobic capacity on the three test days.

Measured (spirometry) and estimated (lactate/ERGONIZER^®^) *V*O_2max_ showed correlations of ICC_3.1_ = 0.91; SEM = 1.62 ml kg^−1^ min^−1^ and ICC_3.1_ = 0.91; CV = 4.98 %, respectively (Table 6; Fig. 4).

**Table 6:** Relative and absolute reliability of maximal aerobic capacity (*V*O_2max_)

Parameter	Pooled mean ± SD	Relative reliability	Absolute reliability	
		ICC_3.1_	SEM	CV
*V*O_2max_ spirometry (ml kg^−1^ min^−1^)^a^	54.7 ± 5.4	0.91	±1.62	±2.97
*V*O_2max_ lactate (ml kg^−1^ min^−1^)^b^	49.3 ± 7.4	0.91	×/÷1.05	×/÷4.98

*SD* standard deviation, *ICC* intraclass correlation, *SEM* standard error of measurement, *CV* coefficient of variation coefficient, “±” error in absolute form, “×/÷” error as ratio, based on log transformed data.

^a^Measured directly using spirometry.

^b^Estimated from lactate values using ERGONIZER^®^ software.

## Discussion

This study investigated the test–retest reliability of the current ESA AFA. It is the first time that this battery of tests has been assessed for their reliability, which is crucial for the future implementation of the AFA with ESA astronauts, to provide relevant feedback in relation to fitness performance and post-flight recovery from exposure to µG.

ESA’s AFA concept of performing physical fitness evaluations wherever ESA crew members are located for training or missions, requires the use of local non-portable hardware (Smith machine, treadmill, stationary gas analysis), which potentially decreases data reliability and comparability between tests performed in different locations. However, this is unavoidable until testing hardware is harmonised across all space agencies or all ESA astronauts are, without exception, assessed in one location, neither of which will happen in the near future. As such, the resulting imprecision needs to be accepted and robust assessment methods must be prioritised over sensitive or unreliable tests.

Although there is considerable variability in the data (ICC_3.1_ ranging from 0.03 to 0.99), 28 of the 41 variables demonstrate reliability above 0.6 (Table 7), which is considered marginally acceptable for occupational field testing that is subject to multiple limitations and over long time periods [24]. For operational purposes, it is important to demonstrate that the elements of the fitness assessment remain at acceptable levels of reliability under different conditions. Good correlations were shown for height, body mass and percentage body fat, which were expected. Hip flexibility (Thomas Test and Sit-and-Reach), muscle strength (1 RM, handgrip and core strength) and *V*O_2max_ (both measured and estimated) also demonstrated acceptable reliability, suggesting that, as long as sufficient standardisation is implemented, traditional assessment methods are satisfactory for physical performance measurements [1, 25–29].

**Table 7:** All parameters ranked (highest to lowest) by relative (ICC_3.1_) reliability

Parameter	ICC_3.1_	SEM	CV
Height (cm)	0.99	±0.23	±0.13
Body mass (kg)	0.99	±0.73	±0.95
1 RM bench press (kg)	0.99	±2.48	±2.84
Sit-and-Reach (cm)	0.99	±1.20	±4.41
Handgrip strength R (kg)	0.97	±1.96	±3.87
Handgrip strength L (kg)	0.95	±2.50	±5.22
1RM Squat (kg)	0.94	±8.67	±6.4
*V*O_2max_ (Spirometry) (ml kg^−1^ min^−1^)^a^	0.91	±1.62	±2.97
*V*O_2max_ (lactate) (ml kg^−1^ min^−1^)^b^	0.91	×/÷1.05	×/÷4.98
Body fat (%)	0.89	±1.80	±15.81
Core strength ventral (s)	0.89	±12.66	±11.00
Core strength lateral (s)	0.86	±7.90	±16.24
Thomas test right (°)	0.85	±2.69	±11.63
RFD CMJ (N s^−1^)	0.85	×/÷1.21	×/÷21.18
RFD SLJ-R (N s^−1^)	0.85	±1.20	±20.10
Path length L4 (mm)	0.80	×/÷1.25	×/÷24.51
Max height DJ (m)	0.79	±0.03	±11.67
Core strength dorsal (s)	0.78	±1.11	±10.71
Max height SJ (m)	0.76	×/÷1.10	×/÷9.76
RFD SJ (N s^−1^)	0.74	×/÷1.24	×/÷24.25
RSI (m s^−1^)	0.73	±0.15	±13.03
RFD SLJ-L (N s^−1^)	0.69	×/÷1.32	×/÷31.98
RFD DJ (N s^−1^)	0.65	×/÷1.44	×/÷43.76
Path length L3 (mm)	0.63	±263.47	25.42
max height CMJ (m)	0.63	×/÷1.14	×/÷14.44
Thomas test left (°)	0.63	±4.12	±16.63
Path length L6 (mm)	0.62	±202.95	±21.55
Path length L5 (mm)	0.61	×/÷1.26	×/÷26.37
Sway area L4 (mm^2^)	0.6	±24.24	±53.35
Sway area L5 (mm^2^)	0.59	×/÷1.71	×/÷70.75
Path length L2 (mm)	0.59	±63.23	±18.24
Sway area L3 (mm^2^)	0.58	×/÷1.66	×/÷66.15
Sway area L2 (mm^2^)	0.57	×/÷1.37	×/÷36.75
Average rotation speed L8 (°/s)	0.55	×/÷1.32	×/÷32.38
Average rotation speed L7 (°/s)	0.54	×/÷1.43	×/÷42.69
Max height SLJ-R (m)	0.51	±0.02	±13.09
Sway area L6 (mm^2^)	0.39	×/÷1.90	×/÷89.71
Average rotation speed L10 (°/s)	0.35	±0.85	±28.21
Path length L1 (mm)	0.21	±24.53	±28.94
max height SLJ-L (m)	0.14	×/÷1.14	×/÷14.76
Sway area L1 (mm^2^)	0.07	×/÷12.20	×/÷54.33
Average rotation speed L9 (°/s)	0.04	±1.38	±42.14

“±” absolute error based on raw data, *CMJ* countermovement jump, *SJ* squat jump, *SLJ-R/L* single-leg jump right/left leg, *DJ* drop jump, *RFD* rate of force development, *RSI* reactive strength index, *L* Level, *SD* standard deviation, *ICC* intraclass correlation coefficient, *SEM* standard error of measurement, *CV* coefficient of variation, “±” error in absolute form, “×/÷” error as ratio, based on log transformed data.

^a^Measured directly using spirometry.

^b^Estimated from lactate values using ERGONIZER^®^ software.

Sit-and-Reach hip flexibility showed a high correlation (ICC_3.1_ = 0.99), which is consistent with previous studies of this field-based test [25]. For longer test intervals, which are the reality for astronauts, greater differences in flexibility might occur. Thus, given its high reliability and relevance for returning astronauts—who demonstrate reduced flexibility after landing (unpublished data from ESA astronauts)—and for health in general, this finding supports maintaining this measurement in the AFA battery.

The modified Thomas Test assessing hip extension flexibility showed lower correlations compared to those reported in the literature [26–28], although are still considered acceptable. Contrary to our study, a goniometer was used in these studies, which may provide better precision compared with an inclinometer, which we chose for reasons of time efficiency. To obtain reliable results, it appears advisable for measurements to be implemented by a well-trained examiner [30], and the experimenter in this study (and ESA staff members who conduct the AFA) was trained to perform the test. As such, to improve the quality of this test, the identification and use of a time-efficient goniometer setup should be prioritised.

The high reliability seen for muscular strength assessments are encouraging, and reflect the careful manner in which they were implemented. Measurement bias was detected with the *F* test for the estimate of squat 1 RM which might reflect a training effect; although all participants had experience with weight lifting, not all of them performed weekly resistance training, and thus potentially “profited” from this additional training stimulus. A stable form when performing a maximum effort squat and bench press evaluation is required for providing consistent values [17, 31]. The simple, multiple repetition estimation method used in the present study appears appropriate for application with astronauts, who, in preparation for and during space missions, perform daily exercises including the squat and bench press, by default, develop sufficient technical skills and are considered sufficiently experienced weight lifters to produce reliable data.

Handgrip strength appears a fast, simple and reliable measure, and thus warrants being maintained in the AFA test battery. For core strength, based on the present findings, one might consider removing the dorsal and lateral position tests from the AFA, and only keeping the ventral position, although all three tests showed acceptable reliability. Although it was not assessed in this study, a trend for decreased performance was visible in the lateral and dorsal position data. This might be related to shoulder or hamstring fatigue occurring prior to onset of trunk muscle fatigue and possibly as a result of the order of testing (ventral always first). Reducing the core test to a single position would enhance time efficiency and likely reduce fatigue effects, so future work in this area should focus on identifying possible carryover effects with multiple tests and which of the three tests best reflects overall core muscle function. If only one test were to be selected, the ventral position might be preferable, as it is more stable and potentially less fatiguing that the others, as it places considerable load on other muscle groups (e.g. shoulders, hamstrings).

Balance and jump tests both show marked variation in measured values between test days, with ICC_3.1_ ranging from 0.14 to 0.85 for jumps and 0.04 to 0.80 for balance, whereas the highest value was shown for the countermovement jump RFD and for balance COP path length L4 (both legs, head tilted back), respectively. Although very reliable, the measurement of static head tilt back (L4) balance capability might not be the strongest parameter for assessing the quality of sensory organisation [32]. The test may still show high reliability without being associated with high validity, which needs to be further investigated.

For balance tests, reliability above ICC_3.1_ = 0.6 was found for COP path length on Level 3–6 on the pressure plate (ICC_3.1_ = 0.61–0.80), thus path length appeared the more reliable parameter compared to COP sway area (ICC_3.1_ = 0.06–0.60), which is consistent with the literature [33, 34]. The lowest reliability was seen for balance tasks on the balance board (rotation velocity L8, L7, L10, L9) (ICC_3.1_ = 0.04–0.55), although both sway area (ICC_3.1_ = 0.07) and path length (ICC_3.1_ = 0.21) at L1 on the pressure platform also demonstrated low values. Given the relative simplicity of the task (both legs, eyes closed), this was surprising, but, being the first balance task each time, it could be related to an initial familiarisation effect (comparable to a warm-up). A repetition of the first task might have removed/reduced this effect, although such a strategy has not been reported in other studies, and subjects in the present study did complete the test during their familiarisation session prior to the study itself.

Ten out of 16 balance parameters displayed ICCs below 0.6, which questions their suitability for inclusion in the AFA. The high random error observed in the present study, despite standardisation of positioning, time and abort criteria, appears a common problem with balance assessments [35], and the influence factors have not been fully identified [34, 36]. Environmental interferences, such as noise or visual stimuli, possibly distracting the subject or day to day changes [1, 37], may affect results, but are difficult to quantify and sometimes unavoidable in field testing. Other studies [35, 36, 38–40] report that balance performance and the reliability of results also vary with trial duration. The task duration of 15 s in the present study might, therefore, be too short and could be extended [24] and, for time efficiency, the application of a smaller number of longer duration tasks and/or more trials with each task might be a better approach.

A high sampling rate of 100 Hz increases the sensitivity of pressure plate measurements and may contribute to higher variance as more data points are tracked [34]. A high sampling rate alone, however, does not seem to increase variance, as shown by Muehlbauer et al. [36], who, applying 400 Hz, obtained good reliability for intra- and inter-session sway area (ICC >0.77 and 0.87) with male participants [36]. Thus, modification of sampling rate may be one, but likely not the only solution for improving reliability in our test setup. A reduction of sampling rate would not affect assessment time, but potentially measurement precision. Sampling rate and test sensitivity may, however, be unrelated, and to obtain certainty, the effect of modifying this parameter would need to be demonstrated with the given test protocol.

The implementation of a balance assessment on an instrumented balance board may well be a novel strategy and a review of the literature revealed no published studies using such a device. The test was included in the battery of balance tests to provide a level of difficulty sufficient to challenge well-trained, younger astronauts in their annual assessments. However, given the low reliability seen in the data, it is possible that the test was simply too difficult to achieve consistent performance. As such, as the primary goal of the AFA is to detect changes in ESA astronaut performance, the removal of the balance board tests should, therefore, be considered.

In the context of astronaut testing, the variability or measurement error should not mask real adaptation effects seen after spaceflight to be a meaningful test. At this stage we cannot confirm that this is the case here, and thus either modification (increase of duration and/or number of trials, modification of sampling rate) or elimination of some balance tasks (e.g. on balance board) should be considered.

Microgravity is known to impact neuro-muscular control mechanisms [10–12], which may affect astronauts’ post-flight muscle power development. The double-legged jump tests showed good reliability as has been reported previously [41, 42], and thus should produce meaningful post-flight data. A marked variation in jump values was observed, showing low reliability for SLJ max height for SLJ-L (ICC_3.1_ = 0.14), and SLJ-R (ICC_3.1_ = 0.51), although all other jump parameters showed ICCs higher than 0.6. For small samples like ours, the mean of all trials could be used instead of the single best effort, to smoothen samples with artefacts. Low reliability in the SLJ may be also related to the technical difficulty of performance, which requires considerable balance. Jump performance variation decreases with increased jumping experience [41, 43, 44], although large variation in vertical jump mechanical variables measured on force platforms occur even in highly trained athletes, and may thus affect reliability [41], and this could be an even bigger factor in astronauts, who are not professional athletes and cover a wide age range (27–60 years). With low reliability in our test population and the potential for even greater variation due to post-flight balance issues experienced by astronauts (and thus also safety concerns), our data suggest that the SLJ tests should be considered for removal from the AFA test battery.

Spirometry and lactate assessments (either alone or in combination) during treadmill running are established measurements for fitness evaluation [45–48]. An estimate and direct measurement of *V*O_2max_ from lactate measurements and spirometry produced high and comparable reliability, and thus appear appropriate for operational use with ESA astronauts. Based on the good reliability results with the ERGONIZER^®^ software, this suggests that it could be used independently from spirometry on the occasions when the spirometry equipment is not available for the AFA to make a direct measurement. We did not assess redundancy between both methods, although this would be useful and should be considered for future evaluation.

There are a number of limitations to this study. We acknowledge that the number of subjects is low, which is a result of the currently limited capacity of ESA staff members to take time away from their operational support roles to perform research activities, as well as to use the test equipment—that is required for performing the AFA with ESA crew members—for non-operational purposes. This limitation was mitigated through the implementation of thorough statistics, including screening data for normality and homoscedasticity, to adequately assess measurement bias and reliability. However, the observed measures of reliability should be treated only as provisional and final decisions as to whether tests should be used in the AFA, modified or removed, should not be taken until each test has been investigated in the respective occupational group, with a larger sample size and in the operational context.

The complexity and length of the test battery and the study conditions may lead to limitations. The pre-set time limitations, hardware portability and the amount of tests to be covered within a 2-h time slot per subject reduce the ability to perform higher standard laboratory assessments and multiple repetitions to increase reliability.

No inter-equipment reliability assessment was performed as part of this study. Currently, the AFA has been conducted at three different sites: the European Astronaut Centre (EAC) in Cologne, Germany, NASA Johnson Space Centre (JSC) in Houston, USA and Gagarin Cosmonaut Training Centre (GCTC) (“Star City”), near Moscow in Russia. European Astronaut Centre equipment is transported for AFAs in Russia, so inter-equipment issues are not an issue in these instances, but they could be when the AFA is conducted at JSC, where a duplicate set of equipment is stored and used. However, as all of the hardware/software used is commercial-off-the-shelf, the variation in construction and performance of different units is likely to be small and thus the influence of inter-equipment variability minimal. Furthermore, although for any individual astronaut, AFAs are set up and conducted in their entirety by one person, three people are qualified to administer the AFA and, it is possible that tests may not be conducted by the same person (e.g. due to illness). As such, inter-observer variation might be an issue for the AFA. We did not address this issue in the present study, but it will be the subject of future investigations.

Although learning and fatigue effects were minimised through weekly sessions, allowing for sufficient recovery and reducing short-time memory, they may have still occurred (e.g. trends in squats or handgrip strength) in the analysed data set. Additional familiarisation sessions or trials prior to measurement could have mitigated this effect, but increased time demands. The testing environment and procedures were standardised to the maximal possible extent with the intention to minimise systematic bias, to identify the random error of measurement, and to ultimately allow a distinction from real performance changes. However, not all external sources of noise or distraction could be fully eliminated nor their effect on results clearly quantified in the data.

## Conclusions

Measurement of height, body mass and percentage body fat, hip flexion/extension, muscular strength (handgrip, core strength and repetition maximum for squat and bench press), double-legged jumps and balance parameters on the pressure plate appear, with minor adjustments enhancing precision, to be adequate for operational implementation of the AFA in the “field test” conditions required for human space flight. Balance tasks implemented on the spherical balance board and single-leg jumps did not demonstrate sufficient reliability, revealing high random error, which could potentially mask effects of µG on astronauts returning from missions. Given practical considerations of operational implementation, mainly those of time constraints, safety aspects, high data complexity and low reliability, and in view of yet undetermined occupational relevance, a comprehensive re-design considering shortening and simplification of the balance protocol is recommended. For similar reasons, the single-leg jumps should be considered for elimination from the battery. High standardisation of procedures should be targeted to mitigate the impact of external factors. Overall the other AFA elements showed acceptable reliability, requiring minor corrections, for continued operational use and further development in the given conditions of space medicine applied in European space flight.

## Abbreviations

AFA: Astronaut Fitness Assessment
ANOVA: analysis of variance
CMJ: countermovement jump
COP: centre of pressure
CV: coefficient of variation
DJ: drop jump
EAC: European Astronaut Centre
ESA: European Space Agency
GRF: ground reaction force
ICC: intraclass correlation coefficient
ISS: International Space Station
L: Level
RFD: rate of force development
RM: repetition maximum
RSI: reactive strength index
SEM: standard error of measurement
SJ: squat jump
SLJ-R/L: single-leg jump right/left leg
*V*O_2max_: maximal oxygen uptake
µG: microgravity

## References

[1] Deakin JM, Pelot R, Eng P, Smith JT, Weber CL (2000). Development and validation of canadian forces minimum physical fitness standard (MPFS 2000).

[2] Serra C, Rodriguez MC, Delclos GL, Plana M, Gomez Lopez LI, Benavides FG (2007). Criteria and methods used for the assessment of fitness for work: a systematic review. Occup Environ Med.

[3] Trappe S, Costill D, Gallagher P, Creer A, Peters JR, Evans H (2009). Exercise in space: human skeletal muscle after 6 months aboard the International Space Station. J Appl Physiol (1985).

[4] Gopalakrishnan R, Genc KO, Rice AJ, Lee SM, Evans HJ, Maender CC (2010). Muscle volume, strength, endurance, and exercise loads during 6-month missions in space. Aviat Space Environ Med.

[5] Shackelford LC, LeBlanc AD, Driscoll TB, Evans HJ, Rianon NJ, Smith SM (2004). Resistance exercise as a countermeasure to disuse-induced bone loss. J Appl Physiol (1985).

[6] Smith SM, Heer MA, Shackelford LC, Sibonga JD, Ploutz-Snyder L, Zwart SR (2012). Benefits for bone from resistance exercise and nutrition in long-duration spaceflight: evidence from biochemistry and densitometry. J Bone Miner Res.

[7] Moore AD, Lee SMC, Stenger MB, Platts SH (2010). Cardiovascular exercise in the U.S. space program: past, present and future. Acta Astronaut.

[8] Convertino V, Sandler H (1995). Exercise countermeasures for spaceflight. Acta Astronaut.

[9] McPhee JC, Charles JB (2009) Human health and performance risk at space exploration missions. Evidence reviewed by the NASA Human Research Program. NASA SP-2009-3405

[10] Wood SJ, Loehr JA, Guilliams ME (2011). Sensorimotor reconditioning during and after spaceflight. NeuroRehabilitation.

[11] Paloski WH, Reschke MF, Black FO, Doxey DD, Harm DL (1992). Recovery of postural equilibrium control following spaceflight. Ann NY Acad Sci.

[12] Reschke MF, Bloomberg JJ, Harm DL, Paloski WH, Layne C, McDonald V (1998). Posture, locomotion, spatial orientation, and motion sickness as a function of space flight. Brain Res Brain Res Rev.

[13] Medical Evaluation Documents (MED) Volume A (2014) Medical standards for ISS crew members. Rev 3.3 SSP 50667

[14] Harvey DG (1998). Assessment of the flexibility of elite athletes using the modified Thomas test. Br J Sports Med.

[15] Tschopp M (2003). Manual Leistungsdiagnostik Kraft.

[16] Brzycki M (1993). Strength testing: predicting a one-rep max from reps to fatigue. J Phys Educ Recreat Dance.

[17] Niewiadomski W, Laskowska D, Gasiorowska A, Cybulski G, Strasz A, Langfort J (2008). Determination and prediction of one repetition maximum (1 RM): safety considerations. J Hum Kinet.

[18] Verdonck A, Wilke C, Froboese I, Nellessen G (1998). Screeningverfahren. Training in der Therapie: Grundlagen und Praxis.

[19] V/CI (1999) Kistler Force Plate Formulae. http://isbweb.org/software/movanal/vaughan/kistler.pdf. Accessed 20 May 2015

[20] Bruce RA, Cooper MN, Gey GO, Fisher LD, Peterson DR (1973). Variations in responses to maximal exercise in health and in cardiovascular disease. Angiology.

[21] Atkinson G, Nevill AM (1998). Statistical methods for assessing measurement error (reliability) in variables relevant to sports medicine. Sports Med.

[22] Hopkins WG (2000). Measures of reliability in sports medicine and science. Sports Med.

[23] Weir JP (2005). Quantifying test–retest reliability using the intraclass correlation coefficient and the SEM. J Strength Cond Res.

[24] Burnstein BDS, Russel J, Shrier I (2011). Reliability of fitness tests using methods and time periods common in sport and occupational management. J Athl Train.

[25] Atamaz F, Ozcaldiran B, Ozdedeli S, Capaci K, Durmaz B (2011). Interobserver and intraobserver reliability in lower-limb flexibility measurements. J Sports Med Phys Fit.

[26] Dennis RJ, Finch CF, Elliott BC, Farhart PJ (2008). The reliability of musculoskeletal screening tests used in cricket. Phys Ther Sport.

[27] Glanzman AM, Swenson AE, Kim H (2008). Intrarater range of motion reliability in cerebral palsy: a comparison of assessment methods. Pediatr Phys Ther.

[28] Gabbe BJ, Bennell KL, Wajswelner H, Finch CF (2004). Reliability of common lower extremity musculoskeletal screening tests. Phys Ther Sport.

[29] Espana-Romero V, Ortega FB, Vicente-Rodriguez G, Artero EG, Rey JP, Ruiz JR (2010). Elbow position affects handgrip strength in adolescents: validity and reliability of Jamar, DynEx, and TKK dynamometers. J Strength Cond Res.

[30] Bartlett MD, Wolf LS, Shurtleff DB, Stahell LT (1985). Hip flexion contractures: a comparison of measurement methods. Arch Phys Med Rehabil.

[31] Ritti-Dias RM, Avelar A, Salvador EP, Cyrino ES (2011). Influence of previous experience on resistance training on reliability of one-repetition maximum test. J Strength Cond Res.

[32] Jain V, Wood SJ, Feiveson AH, Black FO, Paloski WH (2010). Diagnostic accuracy of dynamic posturography testing after short-duration spaceflight. Aviat Space Environ Med.

[33] Maribo T, Stengaard-Pedersen K, Jensen LD, Andersen NT, Schiottz-Christensen B (2011). Postural balance in low back pain patients: intra-session reliability of center of pressure on a portable force platform and of the one leg stand test. Gait Posture.

[34] Raymakers JA, Samson MM, Verhaar HJ (2005). The assessment of body sway and the choice of the stability parameter(s). Gait Posture.

[35] Ruhe A, Fejer R, Walker B (2010). The test-retest reliability of centre of pressure measures in bipedal static task conditions—a systematic review of the literature. Gait Posture.

[36] Muehlbauer T, Roth R, Mueller S, Granacher U (2011). Intra and intersession reliability of balance measures during one-leg standing in young adults. J Strength Cond Res.

[37] Moghadam M, Ashayeri H, Salavati M, Sarafzadeh J, Taghipoor KD, Saeedi A (2011). Reliability of center of pressure measures of postural stability in healthy older adults: effects of postural task difficulty and cognitive load. Gait Posture.

[38] Doyle TL, Newton RU, Burnett AF (2005). Reliability of traditional and fractal dimension measures of quiet stance center of pressure in young, healthy people. Arch Phys Med Rehabil.

[39] Le Clair K, Roach C (1996). Postural stability measures: what to measure and for how long. Clin Biomech.

[40] Lin D, Seol H, Nussbaum MA, Madigan ML (2008). Reliability of COP-based postural sway measures and age-related differences. Gait Posture.

[41] Moir GL, Garcia A, Dwyer GB (2009). Intersession reliability of kinematic and kinetic variables during vertical jumps in men and women. Int J Sports Physiol Perform.

[42] Ditroilo M, Forte R, McKeown D, Boreham C, De Vito G (2011). Intra- and inter-session reliability of vertical jump performance in healthy middle-aged and older men and women. J Sports Sci.

[43] Makaruk H, Czaplicki A, Sacewicz T, Sadowski J (2014). The effects of single versus repeated plyometrics on landing biomechanics and jumping performance in men. Biol Sport..

[44] Kurz G, Lang D, Richter A, Schwameder H (eds) (2009) Reliability of drop jump variations in performance diagnostics. In: ISBS—conference proceedings archive

[45] Pfitzinger P, Freedson PS (1998). The reliability of lactate measurements during exercise. Int J Sports Med.

[46] Pivarnik JM, Dwyer MC, Lauderdale MA (1996). The reliability of aerobic capacity (VO_2max_) testing in adolescent girls. Res Q Exerc Sport.

[47] Tanner RK, Fuller KL, Ross ML (2010). Evaluation of three portable blood lactate analysers: Lactate Pro, Lactate Scout And Lactate Plus. Eur J Appl Physiol.

[48] Vickers RRJ (2003) Measurement error in maximal oxygen uptake tests. Naval Health Research Center, San Diego. Report No.: 04-03

